# Implementation of an “opt-out” tobacco treatment program in six hospitals in South Carolina

**DOI:** 10.1186/s12913-024-11205-7

**Published:** 2024-06-17

**Authors:** K. Michael Cummings, Vincent Talbot, Avery Roberson, Asia A. Bliss, Emily Likins, Naomi C. Brownstein, Stephanie Stansell, Demetress Adams-Ludd, Bridget Harris, David Louder, Edward McCutcheon, Rami Zebian, Alana M. Rojewski, Benjamin A. Toll

**Affiliations:** 1 Department of Psychiatry and Behavioral Sciences, HCC Tobacco Control Program, Hollings Cancer Center, Medical University of South Carolina, 86 Jonathan Lucas Street, Charleston, SC 29425 USA; 2 TelASK Inc, Ottawa, Canada; 3 University of Pikeville, Kentucky College of Osteopathic Medicine, Pikeville, USA; 4 Department of Public Health Sciences, Medical University of South Carolina, Charleston, USA; 5 Department of Population Health, Medical University of South Carolina, Charleston, USA; 6 MUSC Health Alliance, Medical University of South Carolina, Charleston, USA; 7 MUSC Health Lancaster Division, Lancaster, USA; 8 MUSC Health Florence Division, Florence, USA

**Keywords:** Cigarette smoking, Tobacco cessation, Hospital, Health services research, Nicotine addiction, Population health

## Abstract

**Objective:**

Describe the screening, referral, and treatment delivery associated with an *opt-out* tobacco treatment program (TTP) implemented in six hospitals varying in size, rurality and patient populations.

**Methods:**

Between March 6, 2021 and December 17, 2021, adult patients (≥ 18 years) admitted to six hospitals affiliated with the Medical University of South Carolina were screened for smoking status. The hospitals ranged in size from 82 to 715 beds. Those currently smoking were automatically referred to one of two tobacco treatment options: 1) Enhanced care (EC) where patients could receive a bedside consult by a trained tobacco treatment specialist plus an automated post-discharge follow-up call designed to connect those smoking to the South Carolina Quitline (SCQL); or 2) Basic care (BC) consisting of the post-discharge follow-up call only. An attempt was made to survey patients at 6-weeks after hospitalization to assess smoking status.

**Results:**

Smoking prevalence ranged from 14 to 49% across the six hospitals; 6,000 patients were referred to the TTP.The delivery of the bedside consult varied across the hospitals with the lowest in the Charleston hospitals which had the highest caseload of referred patients per specialist. Among patients who received a consult visit during their hospitalization, 50% accepted the consult, 8% opted out, 3% claimed not to be current smokers, and 38% were unavailable at the time of the consult visit. Most of those enrolled in the TTP were long-term daily smokers.Forty-three percent of patients eligible for the automated post-discharge follow-up call answered the call, of those, 61% reported smoking in the past seven days, and of those, 34% accepted the referral to theSCQL. Among the 986 of patients surveyed at 6-weeks after hospitalization quit rates ranged from 20%-30% based on duration of reported cessation and were similar between hospitals and for patients assigned to EC versus BC intervention groups.

**Conclusion:**

Findings demonstrate the broad reach of an *opt-out* TTP. Elements of treatment delivery can be improved by addressing patient-to-staffing ratios, improving systems to prescribe stop smoking medications for patients at discharge and linking patients to stop smoking services after hospital discharge.

**Supplementary Information:**

The online version contains supplementary material available at 10.1186/s12913-024-11205-7.

## Purpose and objectives

Hospitalization provides an opportunity to identify and engage patients who smoke cigarettes to stop smoking [1–4]. Continued smoking can adversely impact clinical outcomes for patients if they persist in smoking [5–7]. In 2012, the joint commission issued the Tobacco Cessation Performance Measure-Set to encourage hospitals to improve documentation and delivery of smoking cessation treatments to patients [8, 9].

We and others have previously demonstrated the effectiveness of implementing a brief inpatient Tobacco Treatment Program (TTP) modeled after the Joint Commission’s (JC) guidelines [10–22]. However, most of the published studies documenting the impact of hospital based smoking cessation interventions are based on short-term research demonstration projects often conducted in large well-resourced health care settings [4, 10]. The reality is most health care is provided in community hospitals where the systematic delivery of smoking cessation interventions is largely absent.

Over the past decade, the health care system of the Medical University of South Carolina (MUSC) has expanded to include the management of multiple hospitals across the state of South Carolina varying in bed size, rurality and patient populations allowing an opportunity to evaluate and compare implementation of the *opt-ou*t TTP in wider range of hospital settings compared to earlier published studies [6, 7, 19, 20].

Herein, we describe and compare the implementation fidelity of an *opt-out* TTP in each of the six hospitals reporting on screening to identify patients who currently smoke cigarettes, referral to the TTP, and treatment delivery to patients. We also report on the post-discharge smoking status of a sample of patients selected from each of the five acute care hospitals conducted 6-weeks after hospitalization as a way to evaluate the effectiveness of the TTP.

## Methods

This is a descriptive study to compare implementation of an *opt-out* TTP in six MUSC hospitals varying in size, rurality and patient populations. At the time this study was initiated in March 2021, MUSC managed eight hospitals – one of which was a children’s hospital (not included in this study), while the other seven hospitals provided care to adult patients (≥ 18 years). One of the seven hospitals was devoted to caring for patients with psychiatric and/or substance use disorders (Charleston Institute of Psychiatry). For the purposes of this study, we have combined Ashely River Tower Hospital and University Hospital in Charleston into a single group for reporting purposes because the same tobacco treatment specialists (i.e., referred to as specialists hereafter) provided care to patients in these two hospitals both of which were located within walking distance of each other.The two acute care hospitals in Charleston are referred to as Charleston Non-Institute of Psychiatry (Non-IOP). The other four acute care hospitals in the study were located in: Chester, Florence, Lancaster, and Marion South Carolina respectively. The six hospitals ranged in size from 82 to 715 beds.

The workflow of the TTP included 4-steps: 1) Screening to identify adult patients who currently smoke cigarettes; 2) Referral of patients to the TTP offering two treatment options: Enhanced care (EC) which consisted of a tobacco treatment specialist bedside consult plus an automated post-discharge follow-up call designed to connect those smoking to the South Carolina Quitline (SCQL) or 2) Basic care (BC) consisting of the post-discharge follow-up call only; 3) Treatment delivery documentations (i.e., offered and accepted); and 4) Follow-up evaluation. Each of these steps are described below.

In step 1 (Screening), between March 6, 2021 and December 17, 2021, adult patients (≥ 18 years) admitted to six hospitals were screened for their cigarette status. EHRs of patients admitted to the hospital were scanned daily to identify patients who currently smoke cigarettes. All of the hospitals in this study utilized Epic™ software to record smoking status and other information about the patient during their hospital stay.

In step 2 (Referral), all adults identified as currently smoking in the EHR were automatically referred to the TTP with the following exceptions: a) patients who did not speak English; b) patients receiving hospice care; and c) patients who had previously been admitted to the hospital and referred to the TTP in the past 6-months. The 6-month referral criterion was put into place to maximize limited resources directed toward enrolling those patients newly identified as currently smoking. Those currently smoking and eligible for the TTP were automatically referred to one of two randomly assigned treatment options: 1) EC or 2) BC. Randomization assignments were carried out in a ratio of approximately 3:1 for every EC to BC patient.

In step 3 (Treatment delivery documentation), we documented the offer and receipt of treatments to patients assigned to either the EC or BC groups. Three full-time specialists provided bedside consults to eligible patients assigned to the EC group in the six hospitals. In the Charleston hospitals the specialist also received additional counseling support from two part-time psychology interns who saw patients approximately two days per week. A second full-time specialist was assigned to see patients in the Florence and Marion Medical Center hospitals, and the third specialist was assigned to see patients in Lancaster and Chester Medical Center hospitals. The bedside consult utilized motivational interviewing to engage with patients. Patients were able to *opt-out* of the bedside consult if they wanted. Consult dispositions were categorized as follows: 1) Received consult; 2) Refused consult; 3) Ineligible for consult because the patient reported not smoking currently (i.e., false positive based on intake screening); and 4) Unavailable for consult when visited in the hospital. Among patients receiving consults, we recorded whether the consult was conducted in-person or by phone and the length of the consult coded as ≤ 10 min or > 10 min. Patients receiving the consult were asked to confirm their smoking status and answer questions about their smoking history, current use of other tobacco products, nicotine dependence, past quit attempts, readiness, and confidence in stopping smoking, all of which were used to create a personalized quitting plan. Specialists were not authorized to prescribe or dispense stop smoking medications to patients, however the specialist could recommend (in Epic™ this is referred to as “pend”) a medication order in the patient’s medical record which could then be acted upon by the attending physician or other members of the care team who were authorized to order or prescribe medications to patients.

All patients who provided a working phone number and were discharged to their home or to assisted living facilities were eligible to receive automated post-discharge IVR follow-up calls starting seven days after hospital discharge with the following exceptions. Patients in the EC group who were visited by a specialist and reported that they did not smoke or that they did not want a consult (i.e., opted out of the consult) were also automatically excluded from receiving the automated post-discharge call. Those patients who did receive the automated call were asked if they had smoked any cigarettes in the past seven days; those answering “yes” were asked if they wanted help to quit smoking or stay quit; those answering “yes” to the second question were asked if they wanted to be transferred to the SCQL, where they could get free stop smoking assistance. Those who answered “yes” were transferred to the SCQL, those answering “no” to an immediate “warm” transfer were asked if they would like the SCQL to call them back at a later time instead. Up to six call attempts were made to reach each patient beginning seven days after discharge from the hospital. If a patient failed to answer any of the calls, then we attempted to reach them again one week later (i.e., 14 days after discharge). For patients eligible to receive automated post-discharge calls, we report on the number who answered the call, the number of current smokers who accepted a referral to the SCQL, and among those referred to the SCQL, the number who received services from the SCQL.

In step 4 (Follow-up evaluation), we attempted to contact patients referred to the TTP six-weeks after discharge from the hospital to their home to assess their satisfaction with the care they received while hospitalized, their smoking status, and efforts made to refrain from and/or stop smoking during and following hospitalization. Follow-up evaluation calls were only made to patients with working telephone numbers who were discharged home. The follow-up evaluation survey excluded patients in the one psychiatric hospital, because the quality improvement study that paid for the 6-week follow-up evaluation survey was limited to patients receiving care in the five acute care hospitals only. For the first three months of the study, we used a random selection process to identify approximately 60 patients per week (i.e. ~ 50%-60% of eligible patients in a week) that would be eligible for the follow-up survey. However, because of the lower than expected completion rate for survey we abandon the random selection process and attempted to reach all eligible patients released for follow-up each week.Overall, there were 3,246 patient deemed eligible for the follow-up survey of whom we secured complete interviews with 986 (i.e., ~ 30%). The completion rate for the survey was similar for patients in each of the five acute care hospitals and for patients assigned to either the EC or BC intervention groups.

Staff conducting follow-up interviews were not involved in providing treatment to patients. To encourage participation in the follow-up evaluation survey, we sent a letter to eligible patients explaining that they had been selected to participate in a brief 10-min phone survey asking them about their recent hospitalization and alerting them to expect a call within 7–10 days. Patients who participated in the survey were given a $10 Amazon e-gift card to compensate them for their time.

### Data sources

Data reported in this paper come from: (1) hospital EHRs; (2) the bedside consult electronic form, which captured information about the patients smoking history and treatment plans; and (3) the six-week phone follow-up evaluation survey conducted using REDCap.

### Ethics approval and consent

Informed consent occurred verbally for the 6-week follow up survey. Consent was obtained from all of the participants that should be informed. All methods were carried out in accordance with relevant guidelines and regulations. The quality improvement study was reviewed by and received ethics clearance through the Medical University of South Carolina IRB Committee (IRB#107,000) with a HIPAA Waiver of Authorization for Research in which approval was granted for: 1) limited protected health information (PHI) in the EHR; and 2) verbal consent for the 6-week telephone follow-up survey. A copy of the study protocol is available from the authors upon request.

EHRs contain information on the patients admission and discharge dates, unit where the patient was located during their hospital stay, demographics (i.e., age, race, sex, type of insurance), smoking status (i.e., never, former, current, unknown), and identifying information such as full name, address, and phone number. Data on daily admissions were extracted from EHRs and deposited via a secure, HIPAA-compliant transfer protocol to the Quit Plan Manager™ system (QPM 5.0, by TelASK Inc, Mount Pleasant, South Carolina, USA) which identified currently smoking patients eligible for the TTP. Each morning, a generated list of inpatient smokers from the prior days’ admissions were presented to the specialist through the QPM secure web-interface that could be accessed using a desktop computer and/or tablet. The interface loaded patients’ identifying information to a task list that informs the specialist where patients were located in the hospital. For patients seen by the specialist, information on the consult was captured on the tablet and securely uploaded to the patient database system. Data from the EHRs were received daily allowing us to update a patient’s smoking status and also detect patients discharged from the hospital and eligibility for receiving automated follow-up calls. Based on the patients’ hospital-assigned medical record number, QPM identified patients who have been readmitted to the hospital within the previous six-months and excluded these patients from re-enrollment in the service. Patients were eligible for re-enrollment in the TTP if readmitted to the hospital after 6-months. In this paper, we only report on the patients’ first admission to the hospital during the reference period. Data from EHRs, bedside consult, and automated calls were captured in separate databases and carefully linked together for analysis via the patients’ medical record number and hospital admission date.

Data on all patients enrolled in the TTP allowed the specialists and clinical chief of the TTP (BAT) to track the status of patients eligible for the service. Weekly reports were generated to track screening, referral, and treatment delivery results for each hospital. The six-week post-discharge follow-up evaluation surveys were tracked separately to ensure sufficient interviews were completed for patients in each of the participating hospitals involved in the quality improvement study.

### Data analysis

Data analyses focus on describing the implementation of the four components of the opt out TTP program: 1) Screening; 2) Referral to the TTP; 3) Treatment delivery of the bedside consult and automated post-discharge follow-up calls designed to link patients to the SCQL; and 4) Follow-up of patients 6-weeks after hospitalization to assess smoking status. The following implementation measures are reported upon for each hospital: (1) the number and proportion of patients screened who endorsed current smoking; (2) among patients reporting current smoking, the proportion of referred to the TTP and assigned to either the EC or BC intervention groups; (3a—EC group only) the proportion visited by a specialist while hospitalized and receiving a bedside consult; (3b – EC and BC groups) the number and proportion eligible to receive a post-discharge follow-up call, the proportion who answered the call, reported currently smoking, and accepted the referral to the SCQL call; and (4) among patients selected for the 6-week follow-up interview, the number and proportion who completed the survey and among survey completers the proportion who reported not smoking at the time of the 6-week follow-up evaluation. Smoking status was defined as a) having not smoked at all since discharge from the hospital (i.e., continuous abstinence); b) not smoking on the day of the follow-up interview (24-h non-smoker prevalence); c) not smoked in the past 7-days (7-day non-smoker prevalence); and d) not smoked in the past 30-days (30-day non-smoker prevalence). For patients who report smoking at the time of the six-week follow-up interview, we also report on the proportion who have made a quit attempt since their hospital stay and the proportion interested in receiving additional assistance to stop smoking.

Frequencies and percentages were reported for categorical variables whereas medians, ranges, means, and standard deviations (SD) were reported for continuously measured variables. All statistical analyses were performed in SAS 9.4™ (SAS Institute, Cary, NC).

## Results

**Fig. 1:**
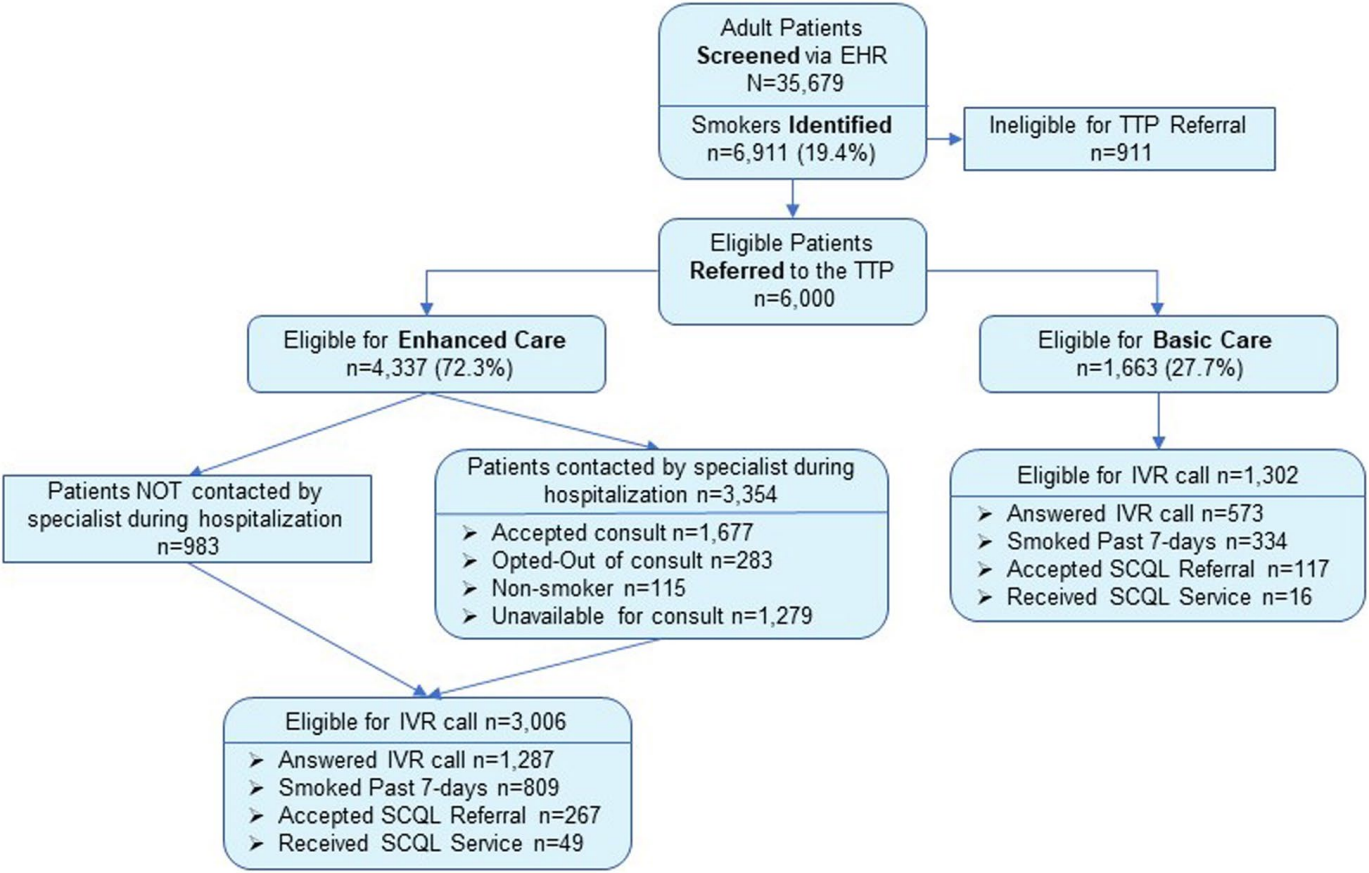
EHR screening, referral, and treatment delivery documentation

Table 1 provides descriptive information on each of the six hospitals and the demographic and health status of current smokers tracked as part of the study. Figure 1 displays the overall workflow of the tracking process from screening patients for smoking status, referral of current smokers to the TTP, and the delivery of treatment interventions to patients assigned to the EC and BC intervention groups.

**Table 1:** Characteristics of the hospitals and patients referred to the tobacco treatment program (TTP)

	Charleston Non-IOP*	**Charleston Institute of Psychiatry (IOP)**	**Florence Medical Center**	**Marion Medical Center**	**Lancaster Medical Center**	**Chester Medical Center**	**Combined**
Type of facility	Acute care	Psychiatric & substance use disorders	Acute care	Acute care	Acute care	Acute care	
Bed size	715	105	124	396	225	82	1647
Number of patients seen during the reference period	21,486	1462	7467	1288	3289	687	35,679
Smoking status							
• Current	2991 (13.9%)	721 (49.3%)	1768 (23.7%)	347 (26.9%)	882 (26.8%)	202 (29.4%)	6911 (19.4%)
• Former	6125 (28.5%)	185 (12.7%)	1833 (24.6%)	275 (21.4%)	883 (26.9%)	196 (28.5%)	9497 (26.6%)
• Never	10,973 (51.1%)	530 (32.3%)	3681 (49.3%)	652 (50.6%)	1503 (45.7%)	271 (39.5%)	17,610(49.4%)
• Unknown	1397 (6.5%)	26 (1.8%)	185 (2.5%)	14 (1.1%)	21 (0.6%)	18 (2.6%)	1661 (4.7%)
Current smokers referred to the TTP							
• Enhanced care (EC)	1761 (58.9%)	474 (65.7%)	1171 (66.2%)	226 (65.1%)	576 (65.3%)	129 (63.9%)	4337 (62.8%)
• Basic care (BC)	668 (22.3%)	177 (24.5%)	431 (24.4%)	88 (25.4%)	241 (27.3%)	58 (28.7%)	1663 (24.1%)
• Not referred	561 (18.8%)	70 (9.7%)	166 (9.4%)	33 (9.2%)	65 (7.4%)	15 (7.4%)	911 (13.2%)
**Characteristics of the patients who currently smoke and were referred to the TTP**							
Average age (years)	51.3	39.3	51.1	54.6	53.1	57.2	50.5
Gender							
• Female	1020 (42.0%)	232 (35.6%)	737 (46.0%)	141 (44.9%)	410 (50.2%)	93 (49.7%)	2633 (43.9%)
• Male	1408 (58.0%)	419 (64.4%)	865 (54.0%)	173 (55.1%)	407 (49.8%)	94 (50.3%)	3366 (56.1%)
• Unknown	1 (0.0%)	-	-	-	-	-	1 (0.0%)
Race							
• Black	822 (33.8%)	224 (34.4%)	640(40.0%)	148 (47.1%)	238 (29.1%)	85 (45.5%)	2157 (36.0%)
• White	1378 (56.7%)	397 (61.0%)	858(53.6%)	141 (44.9%)	541 (66.2%)	89 (47.6%)	3404 (56.7%)
• Other	230 (9.5%)	30 (9.6%)	104 (6.5%)	25 (8.0%)	38 (4.7%)	13 (7.0%)	439 (7.3%)
Veteran Status							
• Yes- active	6 (0.2%)	1 (0.2%)	2 (0.1%)	1 (0.3%)	0 (0.0%)	1 (0.5%)	11 (0.2%)
• Yes- retired	178 (7.3%)	16 (2.5%)	93 (5.8%)	22 (7.0%)	38 (4.7%)	12 (6.4%)	359 (6.0%)
• No	2245 (92.4%)	634 (97.4%)	1507 (94.1%)	291 (92.7%)	779 (95.3%)	174 (93.0%)	5630 (93.8%)
Insurance status							
• Private	557 (22.9%)	111 (17.1%)	285 (17.8%)	30 (9.6%)	114 (14.0%)	19 (10.2%)	1116 (18.6%)
• Medicare	744 (30.6%)	105 (16.1%)	522 (32.6%)	132 (42.0%)	283 (34.6%)	83 (44.4%)	1869 (31.2%)
• Medicaid	492 (20.3%)	139 (21.4%)	407 (25.4%)	66 (21.1%)	210 (25.7%)	31 (16.6%)	1345 (22.4%)
• Other public	305 (12.6%)	31 (4.8%)	128 (8.0%)	32 (10.2%)	66 (8.1%)	18 (9.6%)	580 (9.7%)
• Self-pay	331 (13.6%)	265 (40.7%)	260 (16.2%)	54 (17.2%)	144 (17.6%)	36 (19.3%)	1090 (18.2%)
BMI							
• Underweight	88 (3.6%)	16 (2.5%)	69 (4.3%)	15 (4.8%)	45 (5.5%)	7 (3.7%)	240 (4.0%)
• Normal	517 (21.3%)	205 (31.5%)	376 (23.5%)	58 (18.5%)	232 (28.4%)	52 (27.8%)	1440 (24.0%)
• Overweight	401 (16.5%)	124 (19.0%)	345 (21.5%)	47 (15.0%)	169 (20.7%)	45 (24.1%)	1131 (18.9%)
• Obese	498 (20.5%)	110 (16.9%)	449 (28.0%)	67 (21.3%)	232 (28.4%)	51 (27.3%)	1407 (23.5%)
Length of stay (mean)	5.9 days	9.0 days	4.9 days	4.3 days	3.8 days	3.9 days	5.5 days
Elixhauser mean score	3.0	2.6	2.9	3.4	3.0	2.7	3.0
Charlson mean score	1.7	0.4	1.5	1.6	1.5	1.5	1.4

*Charleston non-IOP includes two acute care hospitals in Chalreston - Ashley River Tower and University Hospital

### Screening

Between March 6th and December 17th, 2021, 35,679 unique patients were admitted to the six hospitals, of whom 19.4% were currently smoking. Smoking prevalence ranged from 14 to 49% with the highest rate of smoking observed among patients admitted to the psychiatric hospital.

### Referral to the TTP

Of the 6,911 patients identified as current cigarette smokers, 6,000 were referred to the TTP and randomized to either EC or BC treatment conditions. Approximately 72% of referred patients in each hospital were assigned to the EC intervention condition while 28% were assigned to the BC intervention group. All 4,337 patients in the EC group were eligible to receive a bedside consult, but only 3,354 (77.3%) received a consult visit from a specialist during their hospitalization. Only home discharged patients with a working phone were eligible to receive an automated post-discharge follow-up call. However, patients in the EC group who reported that they did not smoke and those who opted-out of the bedside consult also were automatically excluded from receiving the automated post-discharge call.

## Treatment delivery

### Bedside consult

Table 2 shows the delivery of the consult to patients in each of the six hospitals. Patients in the three Charleston hospitals had the highest number of referred patients per specialist and the lowest delivery of the beside consults. Among patients who received a consult visit during their hospitalization, 50% accepted the consult, 8% opted out, 3% were ineligible because they reported not smoking, and 38% were unavailable at the time of the consult visit.

**Table 2:** Delivery of the bedside consult to patients

	**Charleston Non-IOP hospitals**	**Charleston IOP hospital**	**Florence hospital**	**Marion hospital**	**Lancaster hospital**	**Chester hospital**	**Combined**
Referred for a consult^a^	1761	474	1171	226	576	129	4337
Referrals per day per specialist	6.0 per day		4.9 per day		2.5 per day		13.4 per day
Contacted by the specialist	1217	363	1049	194	428	103	3354(77.3%)
Accepted the consult	472	58	672	111	289	75	1677(50.0%)
Refused the consult	160	52	65	6	0	0	283 (8.4%)
Not smoking (ineligible)	54	4	42	9	6	0	115 (3.4%)
Unavailable at the time the consult visit	531	249	270	68	133	28	1279(38.1%)

^a^Only patients randomized to the Enhanced Care intervention were eligible to receive a bedside consult

Table 3 shows the characteristics of patients who accepted the consult. Most of the consults were done in-person, although in one hospital 71% of consults were done by phone. About half of the consults were completed in ≤ 10 min, although the time spent counseling varied among the three specialists. The specialist serving patients in the Florence and Marion Medical Center hospitals reporting over 94% of the consults lasting ≤ 10 min, while the other two specialists had the majority of their consults lasting > 10 min.

**Table 3:** Characteristics of patients who received the consult

	**Charleston Non-IOP**	**Charleston IOP**	**Florence**	**Marion**	**Lancaster**	**Chester**	**Combined**
Received consult	**472**	**58**	**672**	**111**	**289**	**75**	**1677**
Encounter type							
• In-person	469 (99.4%)	58 (100%)	635 (94.5%)	93 (83.8%)	169 (58.5%)	22 (29.3%)	1446 (86.2%)
• Phone	1 (0.2%)	0 ( 0.0%)	37 (5.5%)	18 (16.2%)	120 (41.5%)	53 (70.7%)	229 (13.7%)
• Missing	2 (0.4%)	0 ( 0.0%)	0 (0.0%)	0 (0.0%)	0 (0.0%)	0 (0.0%)	2 (0.1%)
Length of consult							
• ≤ 10 min	83 (17.6%)	23 (39.7%)	641 (95.4%)	104 (93.7%)	6 (2.1%)	0 (0.0%)	857 (51.1%)
• > 10 min	375 (79.4%)	33 (56.9%)	29 (4.3%)	7 (6.3%)	282 (97.6%)	74 (98.7%)	800 (47.7%)
• Missing	14 (3.0%)	2 (3.4%)	2 (0.3%)	0 (0.0%)	1 (0.3%)	1 (1.3%)	20 (1.2%)
**Characteristics of those who received the consult **(*n*** = 1677)**							
Average age (years)	51.3	38.5	51.3	54.7	52.5	58.2	54.7
Gender							
• Female	193 (40.9%)	26 (44.8%)	304 (45.2%)	55 (49.5%)	151 (52.2%)	39(52.0%)	768 (45.8%)
• Male	279 (59.1%)	32 (55.2%)	368 (54.8%)	56 (50.5%)	138 (47.8%)	36(48.0%)	909 (54.2%)
Average duration of cigarette use (years)	27.3	19.2	29.4	31.4	31.1	34.8	29.1
Regular use							
• Daily	409 (86.7%)	51 (87.9%)	584 (86.9%)	94 (84.7%)	254 (87.9%)	63 (84.0%)	1455 (86.8%)
• Non-daily	44 (9.3%)	4 (6.9%)	63 (9.4%)	12 (10.8%)	34 (11.8%)	10 (13.3%)	167 (10.0%)
• Missing	19 (4.0%)	3 (5.2%)	25 (3.7%)	5 (4.5%)	1 (0.3%)	2 (2.7%)	55 (3.3%)
Cigs per day^a^	13.5	20.1	13.2	14.4	14.5	11.6	13.8
Time to 1st cig							
• ≤ 5 min	213 (45.1%)	37 (63.8%)	216 (32.1%)	31 (27.9%)	252 (87.2%)	66 (88.0%)	815 (48.6%)
• 6–30 min	81 (17.2%)	8 (13.8%)	140 (20.8%)	32 (28.8%)	28 (9.7%)	7 (9.3%)	296 (17.7%)
• 31–60 min	30 (6.4%)	3 (5.2%)	99 (14.7%)	11 (9.9%)	1 (0.4%)	0 (0.0%)	144 (8.6%)
• > 60 min	115 (24.4%)	6 (10.3%)	176 (26.2%)	30 (27.0%)	5 (1.7%)	2 (2.7%)	334 (19.9%)
• Missing/NA	33 (7.0%)	4 ( 6.9%)	41 (6.1%)	7 (6.3%)	3 (1.0%)	0 (0.0%)	88 (5.3%)
Past quit attempts							
• None	127 (26.9%)	14 (24.1%)	150 (22.3%)	25 (22.5%)	78 (27.0%)	21 (28.0%)	415 (24.8%)
• 1	194 (41.1%)	23 (39.7%)	231 (34.4%)	39 (35.1%)	97 (33.6%)	19 (25.3%)	603 (36.0%)
• > 1	127 (26.8%)	16 (27.6%)	276 (41.1%)	41 (36.9%)	112 (38.8%)	34 (45.3%)	606 (36.1%)
• Missing	24 (5.1%)	5 (8.6%)	15 (2.2%)	6 (5.4%)	2 (0.7%)	1 (1.3%)	53 (3.2%)
Past quit methods^b^							
• Nothing/no help	267 (83.2%)	37 (94.9%)	453 (89.3%)	71 (88.8%)	156 (74.6%)	42 (79.2%)	1025 (84.8%)
• Meds	47 (14.6%)	2 (5.1%)	132 (26.0%)	23 (28.8%)	65 (31.1%)	15 (28.3%)	284 (23.5%)
• E-cig	16 (5.0%)	1 (2.6%)	25 (4.9%)	4 (5.0%)	6 (2.9%)	0 (0.0%)	52 (4.3%)
• Quitline or classes	3 (0.9%)	0 (0.0%)	10 (2.0%)	2 (2.5%)	0 (0.0%)	0 (0.0%)	15 (1.2%)
• Missing	3 (0.9)	0 (0.0%)	9 (1.8%)	1 (0.5%)	2 (1.0%)	1 (1.9%)	16 (1.3%)
Using stop smoking medications while hospitalized	46 (9.8%)	39 (67.2%)	154 (22.9%)	60 (54.1%)	142 (49.1%)	25 (33.3%)	466 (27.8%)
Confidence to quit mean score^c^	7.1	5.9	4.3	4.4	5.8	6.3	5.4
Stage of change							
• No interest	110 (23.3%)	20 (34.5%)	143 (21.3%)	21 (18.9%)	67 (23.2%)	18 (24.0%)	379 (22.6%)
• Not ready, cut down	59 (12.5%)	3 (5.2%)	49 (7.3%)	6 (5.4%)	93 (32.2%)	22 (29.3%)	232 (13.8%)
• Interested in quitting	263 (55.7%)	29 (50.0%)	425 (63.2%)	74 (66.7%)	110 (38.1%)	26 (34.7%)	927 (55.3%)
• Already quit	25 (5.3%)	3 (5.2%)	16 (2.4%)	0 (0.0%)	17 (5.9%)	8 (10.7%)	69 (4.1%)
• Missing	15 (3.2%)	3 (5.2%)	39 (5.8%)	10 (9.0%)	2 (0.7%)	1 (1.3%)	70 (4.2%)

^a^Cigs per day is computed for daily smokers

^b^Reported quit methods is computed based on those who reported one or more quit attempts, more than one method could be reported (i.e., multiple response)

^c^Confidence is scored on a scale ranging from 0 (no confidence) to 10 (high confidence)

The majority of patients who received a consult while hospitalized were male, smoked cigarettes daily, with an average age of 50 years old or greater. Patients from the psychiatric hospital were younger (mean age: 38.5 years) and reported higher levels of cigarette dependence as assessed by cigarettes per day and time to first cigarette of the day. About 75% of patients reported having made a previous attempt to stop smoking. Among those reporting a previous quit attempt, cold-turkey was the most common method reported. About 23% of patients reported past use of stop smoking medications, and only 3% mentioned using an e-cigarette to stop smoking. About 35% of patients reported having strong cravings to smoke while in the hospital, although this varied widely by hospital (i.e., 17% to 62%). Overall, 28% of patients reported receiving stop smoking medications while in the hospital with wide variations seen across the six hospitals (10% to 67%). Patients from the psychiatric hospital were much more likely to report using nicotine replacement medication while in the hospital compared to patients in the acute care hospitals. Readiness and confidence to quit varied among patients in the six hospitals, with about 55% of patients overall contemplating quitting.

### Automated post-discharge call

Table 4 shows the response to the automated IVR calls for patients in the EC and BC interventions for each of the six hospitals. Overall, 72% of patients referred to the TTP were eligible to receive the automated calls. The overall eligibility for the automated post-discharge call was about 13% higher among patients assigned to the BC group when compared to the EC group (78.3% vs. 69.3%), due to the exclusion criteria which, in addition to excluding patients not home discharged, also excluded patients in the EC group who reported not smoking and those who opted-out of the bedside consult.

**Table 4:** Response to the automated calls for patients in the EC and BC for each of the six hospitals

**Intervention group**^**a**^	**Charleston Non-IOP**		**Charleston IOP**		**Florence**		**Marion**		**Lancaster**		**Chester**		**Combined**	
	**EC** *n* = 1761	**BC** *n* = 668	**EC** *n* = 474	**BC** *n* = 177	**EC** *n* = 1171	**BC** *n* = 431	**EC** *n* = 226	**BC** *n* = 88	**EC** *n* = 576	**BC** *n* = 241	**EC** *n* = 129	**BC** *n* = 58	**EC** *n* = 4337	**BC** *n* = 1663
Number and % eligible for the automated post-discharge call^b^ (*n* = 4308)	1179 (67.0%)	518 (77.5%)	340 (71.7%)	141 (79.7%)	809 (69.1%)	336 (78.0%)	148 (65.5%)	72 (81.8%)	431 (74.8%)	190 (78.8%)	99 (76.7%)	45 (77.6%)	3006 (69.3%)	1302 (78.3%)
Number and % reached by IVR call (*n* = 1860)	625 (53.0%)	277 (53.5%)	77 (22.7%)	30 (21.3%)	292 (36.1%)	127 (37.8%)	61 (41.2%)	34 (47.2%)	189 (43.9%)	89 (46.8%)	43 (43.4%)	16 (35.6%)	1287 (42.8%)	573 (44.0%)
Number and % who reported smoking in the past 7 days (*n* = 1143)	373 (59.7%)	143 (51.6%)	52 (67,5%)	20 (69.0%)	177 (66.7%)	68 (53.5%)	41 (67.2%)	31 (91.2%)	135 (71.4%)	60 (67.4%)	31 (72.1%)	12 (75.0%)	809 (62.9%)	334 (58.3%)
Number and % who accepted a transfer or referral to the SCQL (*n* = 384)	107 (28.7%)	47 (32.9%)	13 (25.0%)	5 (25.0%)	70 (39.5%)	27 (39.7%)	12 (35.5%)	11 (35.5%)	52 (38.5%)	24 (40.0%)	13 (41.9%)	3 (25.0%)	267 (33.0%)	117 (35.0%)
Number and percent who received service from the SCQL (*n* = 65)	19 (17.8%)	6 (12.7%)	0 (0.0%)	0 (0.0%)	14 (20.0%)	4 (14.8%)	3 (25.0%)	2 (18.2%)	10 (19.2%)	4 (16.7%)	3 (23.1%)	0 (0.0%)	49 (18.4%)	16 (13.7%)

^a^*EC* Enhanced care, *BC* Basic care

^b^EC patients who reported that they did not smoke and those who opted-out of the bedside consult also were automatically excluded from receiving the automated post-discharge call

Of those eligible to receive the post-discharge call, 1,860 patients were reached yielding an overall response rate of 43% with no significant differences between the EC and BC intervention groups. The response rates to the automated calls ranged from 23% to 53% across the six hospitals, with the lowest response among patients discharged from the psychiatric hospital. Among those responding to the automated calls, 1,143 (62%) reported currently smoking, and of these patients, 384 (34%) accepted the offer of a referral to the SCQL with similar rates of acceptance for patients assigned to the EC and BC groups (33% vs 35%). Data received back from the SCQL found of the 384 patients referred to the SCQL, 17% received services from the SCQL. There were different reasons given for failure to provide service to the referred patients, with the most common reasons being inability to reach patients with follow-up calls (45%), patients not interested in the service (19%), and patients 35% deemed ineligible because they had been enrolled in the SCQL within the past year (36%).

### Overall treatment delivery

Among 4,337 patients assigned to the EC group, 605 (14%) received a bedside consult and answered the automated post-discharge call; 1,072 (25%) received only the bedside consult; 682 (16%) only received and answered the automated post-discharge call; and the remaining 1,978 (45%) did not receive either a consult or responded to the automated post-discharge call. Among patients in the BC group, 573 (35%) received and answered post-discharge call. Overall, of the 6,000 patients referred to the TTP, 2,932 (49%) received either a beside consult and/or post-discharge call. Treatment delivery varied across the six hospitals as noted above.

#### Six-week follow-up evaluation survey

Table 5 summarizes responses to questions about smoking status, the use of stop smoking medications, quit attempts, and interest in receiving additional quitting assistance among those who reported that they still smoke. Self-reported smoking abstinence rates were fairly similar across the five hospitals, with 21% reporting not smoking since discharge from the hospital (continuous abstinence), 31% not smoking on the day of the follow-up survey (24-h point non-smoker prevalence), 24% reporting not smoking in the past 7-days (7-day non-smoker prevalence), and 22% reporting not smoking in the past 30-days (30-day non-smoker prevalence). Overall, 41% of patients reported getting stop smoking medications either during their hospital stay, at the time of discharge from the hospital, or after discharge. Getting stop smoking medications during their hospital stay varied across the five hospitals. In general, the patients from the smaller hospitals were more likely to report using stop smoking medications during and after hospitalization. Among the 682 patients still smoking at the time of the follow-up interview 58% (*n* = 394) reported having made a quit attempt since being discharged from the hospital and 43% reported interest in receiving assistance to stop smoking.

**Table 5:** Six-week post-discharge follow-up evaluation survey outcomes

**Intervention groups**^**a**^	**Charleston Non-IOP**		**Florence**		**Marion**		**Lancaster**		**Chester**		**Combined**	
	**EC**	**BC**	**EC**	**BC**	**EC**	**BC**	**EC**	**BC**	**EC**	**BC**	**EC**	**BC**
Number randomly selected for the follow-up survey	1117	384	723	225	139	41	370	129	85	33	2434	812
Number and % with complete interviews	364 32.6%	131 34.1%	189 26.1%	62 27.6%	50 36.0%	15 36.6%	97 26.2%	37 28.7%	29 34.1%	12 36.4%	729 30.0%	257 31.7%
% continuous non-smoking abstinence	23.1%	18.3%	21.7%	19.4%	28.0%	0.0%	16.5%	24.3%	20.7%	8.3%	22.1%	17.9%
% 24-h non-smoking prevalence	29.7%	29.8%	32.3%	41.9%	40.0%	13.3%	28.9%	29.7%	24.1%	16.7%	30.7%	31.1%
% 7-day non-smoking prevalence	25.8%	22.9%	24.9%	25.8%	28.0%	6.7%	20.6%	24.3%	20.7%	16.7%	24.8%	22.6%
% 30-day non-smokingprevalence	24.2%	19.2%	22.8%	22.6%	28.0%	0.0%	19.6%	24.3%	20.7%	16.7%	23.3%	19.5%
Made a quit attempt^b^	60.6%	52.2%	53.1%	69.4%	60.0%	38.5%	56.5%	57.7%	59.1%	60.0%	58.0%	55.9%
Interested in quit help^b^	39.5%	42.4%	49.2%	30.6%	56.7%	38.5%	53.6%	46.2%	40.9%	50.0%	44.0%	41.2%
Received medications												
• In hospital	30.5%	23.7%	27.5%	29.0%	50.0%	33.3%	49.5%	51.4%	37.9%	16.7%	33.9%	29.2%
• At Discharge	18.1%	14.5%	14.3%	11.3%	24.0%	13.3%	13.4%	27.0%	10.3%	8.3%	16.6%	15.2%
• After discharge	20.1%	24.4%	21.2%	12.9%	24.5%	20.0%	19.6%	37.8%	27.6%	25.0%	20.9%	23.3%
• Any meds	36.5%	37.4%	37.0%	33.9%	54.0%	33.3%	57.7%	62.2%	51.7%	33.3%	41.3%	39.7%
Satisfied with overall hospital care received	97.0%	93.1%	94.7%	90.3%	88.0%	93.3%	93.8%	97.3%	93.1%	100.0%	95.2%	93.4%

^a^*EC* Enhanced care, *BC* Basic care

^b^Restricted to those who answered “Yes” to having smoked today

## Discussion

This study is unique in that it describes the implementation fidelity of an *opt-out* TTP in six different hospitals varying in size, rurality, and patient populations. The United States Department of Health and Human Services recently requested information on strategies to broaden the delivery of evidence-based smoking cessation treatments to those who continue to smoke cigarettes [23]. The findings confirm evidence from other published studies demonstrating the feasibility of using EHRs to efficiently screen patients admitted to the hospital to identify current cigarette smokers and refer them to a TTP [10–22]. In all six hospitals, the vast majority of the patients referred to the TTP were long-term daily smokers, many of whom had tried to quit in the past, with most reporting no prior use of evidence-based smoking cessation treatments. Even in the acute care hospitals, patients identified as current cigarette smokers likely had other psychiatric co-morbidities such as a history of depression, anxiety, and other substance use disorder, especially alcohol use disorder [24].

Overall, 49% of the 6,000 patients referred to the TTP received some type smoking cessation intervention (i.e., consult or automated post-discharge call). The delivery of smoking cessation treatments was uneven across the six hospitals. The lowest rate of delivery for the bedside consult was in the Charleston hospitals which had a higher average combined daily caseload (6.0 referrals per day) compared to Florence and Marion hospitals (4.9 referrals per day), and Lancaster and Chester hospitals (2.5 referrals per day). Our sense is that a single full-time specialist can comfortably handle about 5 referrals per day which represents an annual referral caseload of between 1,825 patients per full-time specialist.

This study also suggests that providing treatment services to psychiatric patients may require a different intervention model than the one we implemented [4]. The psychiatric hospital setting brought forth a different set of challenges when attempting to deliver the bedside consult. Most psychiatric patients while admitted are typically following a set, daily schedule and therefore have a higher likelihood of being unavailable for a consult when a specialist is ready to visit their room. In addition, there are a number of patients who are unavailable for medical reasons for extended periods, overall highlighting the continued need to explore effective cessation models that compliment the dynamics within psychiatric settings. A potentially more effective model for delivering cessation support to patients in a psychiatric hospital setting may involve training existing psychiatric staff to provide the care rather than referring patients to a centralized TTP.

The annual budget for the inpatient TTP across the six hospitals in this study was $380,902. The budget included expenses for three full-time specialist, two part-time psychology interns, clinical oversight, training and equipment for the specialists, and the vendor contract for the Quit Plan Manager™ system which was used for tracking patients from admission to discharge as well as scheduling and executing automated post-discharge calls for patients. With a total of 6,000 patients referred to the TTP over 287 days of the reference period, the average annual number of referrals is estimated to be 7,630 yielding an average annual per patient cost of $51 per referral (i.e., $380,902/7,630 = $51).

The largest barrier we experienced in delivering the bedside consult to patients was actually reaching patients during their admittance. Of the 4,337 patients referred for a bedside consult, 983 (23%) never received a visit from a specialist while hospitalized. Length of hospitalization was strongly associated with who did and did not receive a visit from the specialist. Among those who received a consult visit from a specialist, the average length of stay was longer when compared to patients who did not receive a visit (6.3 days vs 3.3 days, *p* < 0.01).Another relatively large group (38%, *n* = 1,279) that did not get a consult while hospitalized included patients unavailable for the consult at the time the specialist came to their hospital room. The group of patients unavailable for a consult was overrepresented in our psychiatric hospital which as noted above included many patients who do not stay in their hospital room during the day, and thus were unavailable when the specialist came to visit the patient in their room. Excluding the patients from the psychiatric hospital, among patients who were unavailable for a visit, there was an association with length of hospitalization but in the opposite direction than we observed for receiving a specialists’ visit. Patients in the acute care hospitals who received a visit from the specialist and were unavailable for the consult had an average length of stay of 7.5 days compared to 4.7 days (*p* < 0.01) for patients who did receive the consult. Another factor related to patient unavailability for a consult was how sick/ill patients were during their hospitalization. The Elixhauser Comorbidity Index is a measure of overall severity of comorbidities (range: 0 to 16, with higher scores indicating more comorbidities) and the mean score on this index was significantly higher for patient unavailable for a visit compared to those who received a consult (3.3 vs 3.0, *p* < 0.01) [25]. Among patients who received a consult visit while hospitalized, 86% accepted the consult with only 14% opting out, suggesting that patient resistance to tobacco treatment support is not a significant barrier to treatment delivery. The overall *opt-out* rate we observed across the five acute care hospitals combined was similar to the rate we reported in our earlier study of the Charleston non-IOP hospital [19].

The automated calls made to patients 1–2 weeks post hospitalization are a low-cost way to extend smoking cessation services to patients who have relapsed back to smoking after being hospitalized. Forty-three percent of patients were reached with the automated call system using interactive voice recognition. Of those reached, 62% reported that they had returned to smoking within a week or two following their hospital discharge. About one-third of patients who reported currently smoking accepted a referral to the SCQL. Psychiatric inpatients had a lower response to the automated calls, although among those reached, about 25% accepted the referral to the SCQL. A bigger concern with the automated calls was the actual provision of cessation support to patients connected to the SCQL which was much lower than we had expected. Many patients did not answer the follow-up calls from the SCQL or indicated lack of interest in the service after the referral was made, possibly reflecting a lack motivation to quit.

Six-weeks after hospitalization, self-reported smoking abstinence rates ranged from 20–30% depending on how the abstinence rate was defined. Overall, smoking abstinence rates were similar to what we previously reported in our prior study of an *opt-out* inpatient TTP and were similar across the five acute care hospitals [19]. On a positive note, among patients who reported that they were still smoking in our 6-week follow-up survey, 43% expressed interest in receiving additional cessation assistance, reinforcing the benefits of using the hospital admission and immediate post-discharge period as an opportunity to support patients in their journey towards smoking cessation. Finding efficient ways to connect patients who return to smoking after hospitalization to treatment resources is an area that is ripe for future investigation.

Quit rates did not differ for patients in the EC and BC intervention groups indicating a need to strengthen the impact of the brief bedside consult. Other studies have shown that the provision of stop smoking medications to patients at the time of discharge from the hospital is a critical component of an effective stop smoking intervention [10, 22]. While about 40% of patients did report using stop smoking medications during or immediately after discharge from the hospital, the rate of medication use was lower than we had expected it to be among patients receiving the bedside consult where one of the objectives of the consult was to develop and execute a stop smoking treatment plan for patients. The findings from this study reveal that our tobacco treatment specialists achieved only modest success in getting the medical staff to prescribe stop smoking medications to patients upon discharge from the hospital. Only 16.% of patients in the EC group received medication, even though medication treatment recommendations added to majority of the patient’s medical record as a result of their bedside consult. A recent study found that an EHR hard-stop prompt to clinicians prescribing stop smoking medications to patients at the time of admission and discharge significantly increased medication use by patients and post-discharge quit rates [22].

In summary, the findings from this descriptive study demonstrate the broad real-world reach and impact of implementing an *opt-out* TTP in a diverse set of hospitals spread out geographically across the state of South Carolina. Despite these strengths, there are important limitations to this study that should be noted. First, our system of referring patients to the program was restricted to patients classified as currently smoking cigarettes which undoubtedly excludes patients who had recently stopped smoking or are using other types of non-cigarette tobacco products. Second, referrals are based on self-reported smoking status as recorded in the EHR which is not always accurate. Of 3,354 patients in the EC group who were contacted by a specialist, only 3% reported not smoking (i.e., false positives). However, we acknowledge that a likely greater number of patients will fail to accurately report their true current smoking status (i.e., false negative) [26]. Biochemical validation of smoking status represents a potential solution to improving the accuracy of smoking status assessment but was not done as part of screening process because it would have added additional costs to the program [27]. That said, the screening process we employed allowed for repeat checking of a patient’s self-reported smoking status every day while hospitalized so that someone previously screened as not smoking could have their smoking status updated to currently smoking if a change in status was recorded in the EHR. Third, the post-discharge smoking cessation outcomes reported in this paper are based on self-report and only for those who participated in our follow-up survey. If one assumes all non-responders to the follow-up survey were still smoking, the reported quit rates will drop to a range of 6.4% to 9.3% which would markedly reduce the estimated population impact of the program. That said, the same type of limitation would apply to population surveys which are also based on self-reported smoking status assessed at the time of interview (point prevalence) and which also fail to adjust for non-responders [28].

Despite these limitations, this descriptive paper illustrates the feasibility of implementing an *opt-out* TTP for patients seen in a diverse set of hospitals at a relatively low cost per patient referred. Overall, patient acceptance of TTP was high although the impact of the EC intervention was less than we had expected. Our analysis found elements of our treatment delivery can be improved upon through better patient-to-staffing ratios, improved systems to get stop smoking medications prescribed for patients, and improvements in linking patients to stop smoking services after hospital discharge.

### Supplementary Information

Supplementary Material 1.

## Data Availability

A de-identified dataset, data dictionary and copy of our REDCap survey are available upon request made to lead author (K. Michael Cummings, cummingk@musc.edu).

## Abbreviations

TTP: Tobacco Treatment Program
SCQL: South Carolina Quitline
JC: Joint Commission
HER: Electronic Health Record
ATTUD: Association for the Treatment of Tobacco Use and Dependence
Specialist: Tobacco Treatment Specialist
