# Supplementary material for: Implementation of an “opt-out” tobacco treatment program in six hospitals in South Carolina
Source: BMC Health Serv Res. 2024 Jun 17;24:741. doi: 10.1186/s12913-024-11205-7 (PMC11184783; doi:10.1186/s12913-024-11205-7)
Supplement: Supplementary file 1 — Supplementary Material 1. [file 12913_2024_11205_MOESM1_ESM.pdf]

Inpatient 6-Week Follow-up Survey

Please complete the survey below.

Thank you!

PRE-POPULATED DATA

MRN ______________________

Name ______________________

Date of Birth ______________________

age_calc from dob ______________________

Sex
- ◯ Female
- ◯ Male
- ◯ Other
- ◯ Unknown

Admission Date & Time ______________________
(This field is also considered to be the date of enrollment due to randomization on the date of admission. This was advised by the PRC.)

Discharge Date & Time ______________________

Site
- ◯ Charleston - General Hospitals
- ◯ Charleston - Institute of Psychiatry (IOP)
- ◯ Florence
- ◯ Lancaster
- ◯ Marion
- ◯ Chester

Phone Number 1 ______________________

Phone Number 2 ______________________

E-mail Address ______________________

Address ______________________

Date advance letter sent to patient ______________________

CALL LOG

CALL 1

Call Date Time 1

____

Call Outcome 1

◯ Reached: accepted & completed interview
◯ Reached: rescheduled for a more convenient time
◯ Reached: refused interview
◯ Not Reached: left a voicemail, try calling back
◯ Not Reached: did not answer, try calling back
◯ Not Reached: deceased
◯ Not Reached: wrong/bad/out-of-service number

Notes 1

____

CALL 2

Call Date Time 2

____

Call Outcome 2

◯ Reached: accepted & completed interview
◯ Reached: rescheduled for a more convenient time
◯ Reached: refused interview
◯ Not Reached: left a voicemail, try calling back
◯ Not Reached: did not answer, try calling back
◯ Not Reached: deceased
◯ Not Reached: wrong/bad/out-of-service number

Notes 2

____

CALL 3

Call Date Time 3

____

Call Outcome 3

◯ Reached: accepted & completed interview
◯ Reached: rescheduled for a more convenient time
◯ Reached: refused interview
◯ Not Reached: left a voicemail, try calling back
◯ Not Reached: did not answer, try calling back
◯ Not Reached: deceased
◯ Not Reached: wrong/bad/out-of-service number

Notes 3

____

CALL 4

Call Date Time 4 ________________________________

Call Outcome 4
- ◯ Reached: accepted & completed interview
- ◯ Reached: rescheduled for a more convenient time
- ◯ Reached: refused interview
- ◯ Not Reached: left a voicemail, try calling back
- ◯ Not Reached: did not answer, try calling back
- ◯ Not Reached: deceased
- ◯ Not Reached: wrong/bad/out-of-service number

Notes 4 ________________________________

CALL 5

Call Date Time 5 ________________________________

Call Outcome 5
- ◯ Reached: accepted & completed interview
- ◯ Reached: rescheduled for a more convenient time
- ◯ Reached: refused interview
- ◯ Not Reached: left a voicemail, try calling back
- ◯ Not Reached: did not answer, try calling back
- ◯ Not Reached: deceased
- ◯ Not Reached: wrong/bad/out-of-service number

Notes 5 ________________________________

CALL 6

Call Date Time 6 ________________________________

Call Outcome 6
- ◯ Reached: accepted & completed interview
- ◯ Reached: rescheduled for a more convenient time
- ◯ Reached: refused interview
- ◯ Not Reached: left a voicemail, try calling back
- ◯ Not Reached: did not answer, try calling back
- ◯ Not Reached: deceased
- ◯ Not Reached: wrong/bad/out-of-service number

Notes 6 ________________________________

IDENTITY ESTABLISHMENT

Hi, my name is [NAME]. I'm calling on behalf of the Medical University of South Carolina [site], may I speak with [name]?

◯ Yes, they are available
◯ No, they are not available

Can they be reached at this number?

◯ Yes
◯ No

When would be a good time to reach them?

____

Is there a good number to reach them at?

◯ Yes
◯ No/Don't Know. We thank you for your time. NO MORE CALLS. END OF SURVEY. NO COMPENSATION.

What would be a good number to reach them at?

____

On behalf of MUSC Health, we invite you to participate in a brief 10-minute telephone survey. You are being contacted because we are doing a survey to gather information to help us improve the tobacco treatment services we provide to patients seen in our hospitals. The survey will take less than 10-minutes of your time on the phone.

Your participation in this brief survey is completely voluntary and it will have no bearing whatsoever on the healthcare that you receive from MUSC Health. There is a possible risk of loss of confidentiality however, we have taken steps to ensure that your identity will be protected by coding your information. If you choose to participate, you will receive a $10 Amazon e-gift card to compensate you for your time. Your consent to participate in this survey is implied by your verbal consent to proceed with the survey. Can we proceed? (record yes or no)

Are you willing to participate in this survey?

◯ Yes - Great. Let's get started.
◯ No - No problem, we thank you for your time. [No more calls. End of Survey. No Compensation]

1. Were you satisfied with the clinical care you received when you were recently hospitalized at [site]?

◯ Yes
◯ No
◯ I'd rather not say

2. Since you were discharged from [site] on [discharge_date_time] have you had a doctor's visit outside the hospital for medical assistance and/or evaluation?

◯ Yes
◯ No

Was the visit to the doctor something that was expected OR was it due to an emergency?

◯ Expected (i.e., planned)
◯ Emergency

3. Since you were discharged from [site] on [discharge_date_time] have you sought medical care at an emergency room for yourself?

◯ Yes
◯ No

4. Since you were discharged from [site] on [discharge_date_time] have you been readmitted back into a hospital?	◯ Yes<br>◯ No
Was the readmission back into the hospital something that was expected or scheduled in advance or was it due to an emergency?	◯ Expected (i.e., planned)<br>◯ Emergency

Now, I'd like to ask you some questions about your history of smoking cigarettes and services you might have received while you were in the hospital. Ok?

5. My records show you were a cigarette smoker when you were admitted to the hospital. Is that correct?	◯ Yes<br>◯ No - I'm sorry, we got the wrong information. I don't have any more questions for you. Thanks for your time. [End of survey. No Compensation]

6. Approximately how many years altogether have you smoked cigarettes in your lifetime?

◯ 1
◯ 2
◯ 3
◯ 4
◯ 5
◯ 6
◯ 7
◯ 8
◯ 9
◯ 10
◯ 11
◯ 12
◯ 13
◯ 14
◯ 15
◯ 16
◯ 17
◯ 18
◯ 19
◯ 20
◯ 21
◯ 22
◯ 23
◯ 24
◯ 25
◯ 26
◯ 27
◯ 28
◯ 29
◯ 30
◯ 31
◯ 32
◯ 33
◯ 34
◯ 35
◯ 36
◯ 37
◯ 38
◯ 39
◯ 40
◯ 41
◯ 42
◯ 43
◯ 44
◯ 45
◯ 46
◯ 47
◯ 48
◯ 49
◯ 50
◯ 51
◯ 52
◯ 53
◯ 54
◯ 55
◯ 56
◯ 57
◯ 58
◯ 59
◯ 60
◯ 61
◯ 62
◯ 63
◯ 64
◯ 65
◯ 66
◯ 67
◯ 68
◯ 69

	◯ 70<br>◯ Don't Know
7. How old were you when you first started buying packs of cigarettes for yourself?	◯ < 15<br>◯ 15-17<br>◯ 18-21<br>◯ >21
8. Have you smoked a cigarette since you were discharged from the hospital on [discharge_date_time]?	◯ Yes<br>◯ No - Great. [Skip to Q12]
9. Have you smoked any cigarettes today?	◯ Yes<br>◯ No - [Continue to Q10]
How much do you typically smoke per day? [Cigarettes]	______________________________
How many days after you were discharged from the hospital did you first smoke a cigarette, even a puff or two?<br><br>[Skip to Q13]	◯ 1<br>◯ 2<br>◯ 3<br>◯ 4<br>◯ 5<br>◯ 6<br>◯ 7<br>◯ 8<br>◯ 9<br>◯ 10<br>◯ 11<br>◯ 12<br>◯ 13<br>◯ 14<br>◯ 15<br>◯ 16<br>◯ 17<br>◯ 18<br>◯ 19<br>◯ 20<br>◯ 21<br>◯ 22<br>◯ 23<br>◯ 24<br>◯ 25<br>◯ 26<br>◯ 27<br>◯ 28<br>◯ 29<br>◯ 30<br>◯ Don't Know
10. You told me you have not smoked today, but have you smoked any cigarettes in the past 7 days?	◯ Yes<br>◯ No [Continue to Q11]
How many days out of the past 7 have you smoked a cigarette even a puff or two?	◯ 1 out of 7<br>◯ 2 out of 7<br>◯ 3 out of 7<br>◯ 4 out of 7<br>◯ 5 out of 7<br>◯ 6 out of 7

On the days you do smoke how much do you typically smoked per day? [Skip to Q13]	◯ 1<br>◯ 2<br>◯ 3<br>◯ 4<br>◯ 5<br>◯ 6<br>◯ 7<br>◯ 8<br>◯ 9<br>◯ 10<br>◯ 11<br>◯ 12<br>◯ 13<br>◯ 14<br>◯ 15<br>◯ 16<br>◯ 17<br>◯ 18<br>◯ 19<br>◯ 20<br>◯ 21<br>◯ 22<br>◯ 23<br>◯ 24<br>◯ 25<br>◯ 26<br>◯ 27<br>◯ 28<br>◯ 29<br>◯ 30<br>◯ Don't Know
11. You told me you haven't smoked any cigarettes in the past week, but how about in the past month. Have you smoked any cigarettes in the past 30 days?	◯ Yes<br>◯ No [Continue to Q12]

How many days out of the past 30 have you smoked a cigarette even a puff? [Days]

- ◯ 1
- ◯ 2
- ◯ 3
- ◯ 4
- ◯ 5
- ◯ 6
- ◯ 7
- ◯ 8
- ◯ 9
- ◯ 10
- ◯ 11
- ◯ 12
- ◯ 13
- ◯ 14
- ◯ 15
- ◯ 16
- ◯ 17
- ◯ 18
- ◯ 19
- ◯ 20
- ◯ 21
- ◯ 22
- ◯ 23
- ◯ 24
- ◯ 25
- ◯ 26
- ◯ 27
- ◯ 28
- ◯ 29
- ◯ 30
- ◯ Don't Know

On the days you do smoke how much do you typically smoke per day? [Cigarettes per day].

[Skip to Q13]

◯ 1
◯ 2
◯ 3
◯ 4
◯ 5
◯ 6
◯ 7
◯ 8
◯ 9
◯ 10
◯ 11
◯ 12
◯ 13
◯ 14
◯ 15
◯ 16
◯ 17
◯ 18
◯ 19
◯ 20
◯ 21
◯ 22
◯ 23
◯ 24
◯ 25
◯ 26
◯ 27
◯ 28
◯ 29
◯ 30
◯ Don't Know

12. In what month and year did you last smoke a cigarette even a puff or two?

____
(Enter 1st day of the month. The day will be ignored.)

13. Before being admitted to the hospital, did you use...

	Everyday	Some days	Not at all
a) Traditional cigars, cigarillos or filtered cigars?	◯	◯	◯
b) Smokeless tobacco such as moist snuff, snus or chewing tobacco?	◯	◯	◯
c) An electronic cigarette/Vaping device?	◯	◯	◯

14. During your recent hospitalize stay, did you smoke any cigarettes?

◯ Yes - Outside the hospital?
◯ Yes - Sneaked it in my room
◯ Yes - other places
◯ No

15. During your recent hospitalization, did you use an e-cigarette or a vaping device?

◯ Yes - Outside the hospital?
◯ Yes - Sneaked it in my room
◯ Yes - other places
◯ No

16. Did you receive any of the following stop smoking medications while you were hospitalized?

	Yes	No	Don't Know
Nicotine patch?	◯	◯	◯
Nicotine gum?	◯	◯	◯
Nicotine lozenge?	◯	◯	◯
Nicotine inhaler?	◯	◯	◯
Nicotine nasal spray?	◯	◯	◯
A drug called bupropion, sometimes referred to as Zyban or Wellbutrin?	◯	◯	◯
A drug called varenicline, sometimes referred to as Chantix?	◯	◯	◯

17. When you were discharged from the hospital, did your doctor give you any of the following stop smoking medications, including prescriptions?

	Yes	No	Don't Know
Nicotine patch?	◯	◯	◯
Nicotine gum?	◯	◯	◯
Nicotine lozenge?	◯	◯	◯
Nicotine inhaler?	◯	◯	◯
Nicotine nasal spray?	◯	◯	◯
A drug called bupropion, sometimes referred to as Zyban or Wellbutrin?	◯	◯	◯
A drug called varenicline, sometimes referred to as Chantix?	◯	◯	◯

18. Since you were discharged, have you used any of the following stop smoking medications?

	Yes	No	Don't Know
Nicotine patch?	◯	◯	◯
Nicotine gum?	◯	◯	◯
Nicotine lozenge?	◯	◯	◯
Nicotine inhaler?	◯	◯	◯
Nicotine nasal spray?	◯	◯	◯
A drug called bupropion, sometimes referred to as Zyban or Wellbutrin?	◯	◯	◯
A drug called varenicline, sometimes referred to as Chantix?	◯	◯	◯

19. Since you were discharged from the hospital on [discharge_date_time], please tell me if you've had any of the following experiences...

	Yes	No	Don't Know
a) Made a quit attempt?	◯	◯	◯
b) Purchased over-the-counter stop smoking medication like a nicotine patch, gum, or lozenge?	◯	◯	◯
c) Used an electronic cigarette to try to stop smoking?	◯	◯	◯
d) Talked to a health professional about your smoking?	◯	◯	◯
e) Got a prescription from a doctor or pharmacist for medicine to help me stop smoking?	◯	◯	◯
f) Received a phone call from someone at MUSC asking you about your smoking?	◯	◯	◯
g) Talked to someone at the South Carolina Tobacco Quitline?	◯	◯	◯
h) Went to a stop smoking class or clinic?	◯	◯	◯

I just have a few more questions and then we will be done.

20. Does anyone who lives in your household currently smoke cigarettes?

◯ Yes
◯ No - I live alone.
◯ No - No one else smokes cigarettes

21. Would you like any assistance to help you quit or stay quit?

◯ Yes
◯ No
◯ Don't Know

22. Great. I can send an e-referral to the South Carolina Quitline which is a free stop smoking service if you are interested. Is that something you would like me to do?

◯ Yes
◯ No

E-Referral sent?

◯ Yes
◯ Patient is already referred

23. What suggestions, if any, do you have for how [site] can do a better job to help patients stop smoking?

____

24. Thank you for your time. You are eligible for $10 e-gift code from Amazon to compensate you for your time talking with us today. How would you like to receive this e-gift code?

◯ Mail
◯ E-mail
◯ Declined (don't want the gift code)

Address [Address]

____

Final Disposition	◯ Reached: accepted & completed interview<br>◯ Reached: accepted & partially completed interview<br>◯ Reached: refused interview<br>◯ Not Reached: after 6 calls<br>◯ Not Reached: wrong/bad/out-of-service number<br>◯ Reached: Ineligible (deceased)<br>◯ Reached: Ineligible (does not speak English)<br>◯ Reached: Ineligible (underage)<br>◯ Reached: Ineligible (not a smoker)<br>◯ Reached: Ineligible (readmitted to hospital)<br>◯ Other
E-mail [E-mail]	______________________________
Final Disposition Date Time	______________________________
This interview was completed by:	◯ Asia<br>◯ Majayla<br>◯ Other Research Staff
Notes:	______________________________
If Other, please explain:	______________________________
Is the patient a "cigar only" smoker?	◯ Yes<br>◯ No
